# Publisher's Note

**DOI:** 10.1093/femsyr/foac051

**Published:** 2022-11-11

This is Publisher's Note regarding:

- Elena Vanacloig-Pedros, Kaitlin J Fisher, Lisa Liu, Derek J Debrauske, Megan K M Young, Michael Place, Chris Todd Hittinger, Trey K Sato, Audrey P Gasch, Comparative chemical genomic profiling across plant-based hydrolysate toxins reveals widespread antagonism in fitness contributions, *FEMS Yeast Research*, Volume 22, Issue 1, 2022, foac036, https://doi.org/10.1093/femsyr/foac036
- Sebastian A Tamayo Rojas, Eckhard Boles, Mislav Oreb, A yeast-based *in vivo* assay system for analyzing efflux of sugars mediated by glucose and xylose transporters, *FEMS Yeast Research*, Volume 22, Issue 1, 2022, foac038, https://doi.org/10.1093/femsyr/foac038
- Daniel Elias, Nora Toth Hervay, Juraj Jacko, Marcela Morvova, Jr, Martin Valachovic, Yvetta Gbelska, Erg6p is essential for antifungal drug resistance, plasma membrane properties and cell wall integrity in *Candida glabrata*, *FEMS Yeast Research*, Volume 22, Issue 1, 2022, foac045, https://doi.org/10.1093/femsyr/foac045

These three papers were inadvertently assigned to Volume 2021, Issue 1 (2021) instead of Volume 22, Issue 1 (2022). This error, for which the publisher apologizes, has now been corrected.

